# Transient Neonatal Zinc Deficiency Caused by a Heterozygous G87R Mutation in the *Zinc Transporter ZnT-2 (SLC30A2) * Gene in the Mother Highlighting the Importance of Zn^**2+**^ for Normal Growth and Development

**DOI:** 10.1155/2013/259189

**Published:** 2013-09-29

**Authors:** Maria Consolata Miletta, Andreas Bieri, Kristin Kernland, Martin H. Schöni, Vibor Petkovic, Christa E. Flück, Andrée Eblé, Primus E. Mullis

**Affiliations:** ^1^Division of Paediatric Endocrinology, Diabetology and Metabolism and Department of Clinical Research, University Children's Hospital, Inselspital, 3010 Bern, Switzerland; ^2^Department of Dermatology, University of Bern, 3010 Bern, Switzerland

## Abstract

Suboptimal dietary zinc (Zn^2+^) intake is increasingly appreciated as an important public health issue. Zn^2+^ is an essential mineral, and infants are particularly vulnerable to Zn^2+^ deficiency, as they require large amounts of Zn^2+^ for their normal growth and development. Although term infants are born with an important hepatic Zn^2+^ storage, adequate Zn^2+^ nutrition of infants mostly depends on breast milk or formula feeding, which contains an adequate amount of Zn^2+^ to meet the infants' requirements. An exclusively breast-fed 6 months old infant suffering from Zn^2+^ deficiency caused by an autosomal dominant negative G87R mutation in the *Slc30a2* gene (encoding for the zinc transporter 2 (ZnT-2)) in the mother is reported. More than 20 zinc transporters characterized up to date, classified into two families (Slc30a/ZnT and Slc39a/Zip), reflect the complexity and importance of maintaining cellular Zn^2+^ homeostasis and dynamics. The role of ZnTs is to reduce intracellular Zn^2+^ by transporting it from the cytoplasm into various intracellular organelles and by moving Zn^2+^ into extracellular space. Zips increase intracellular Zn^2+^ by transporting it in the opposite direction. Thus the coordinated action of both is essential for the maintenance of Zn^2+^ homeostasis in the cytoplasm, and accumulating evidence suggests that this is also true for the secretory pathway of growth hormone.

## 1. Introduction 

Zinc (Zn^2+^) is an essential mineral, and infants are particularly vulnerable to Zn^2+^ deficiency as they require large amounts of Zn^2+^ for their normal growth and development [1–4]. Further, suboptimal dietary zinc intake is increasingly appreciated as an important public health issue and has been recently reviewed in a workshop organised by the World Health Organization [5]. The rapid growth experienced by term infants during the first months of life, while they are exclusively breastfed, underscores the appropriateness of breast milk. In particularly in the first three months, Zn^2+^ occurs in breast milk unlike iron and copper at much higher concentration [6]. Until recently it was not known why some otherwise healthy and normal nourished and nursing mothers may present with low Zn^2+^ levels in breast milk causing various abnormalities including growth arrest in the baby. Transient neonatal zinc deficiency (TNZD; OMIM number 608118) is one of the disorders well described [7–11] which is characterized by a low level of Zn^2+^ found in serum of exclusively breast-fed infants which occurs due to the defective secretion of Zn^2+^ into mothers milk. This disorder is well distinct, clinically as well as genetically, from the other Zn^2+^-related entity specified as acrodermatitis enterohepathica (AEZ; OMIM number 201100), where the uptake of Zn^2+^ is the inborn error of metabolism [12–14].

Here we describe the clinical case of an exclusively breast-fed 6 months old infant presented to our outpatient clinic suffering from Zn^2+^ deficiency caused by an autosomal dominant negative G87R mutation in the *zinc transporter ZnT2* gene (*SLC30A2*) in the mother. In addition, as the mechanism of Zn^2+^ on growth and development is not well known, we focus further on its impact on growth hormone production/secretion. 

## 2. Case Report/Methods

### 2.1. Case Report

**Figure 1:**
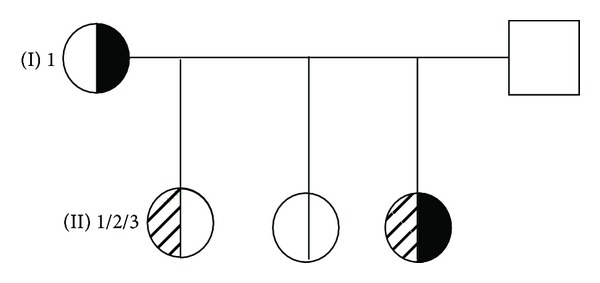
*Pedigree*. Full half circle: heterozygosity G87R SLC30A2; hatched circle: clinical signs during infancy while breastfed. II.1, II.2, and II.3 were breastfed for 3, 1, and 6 months, respectively. II.3 is reported and described.

**Figure 2:**
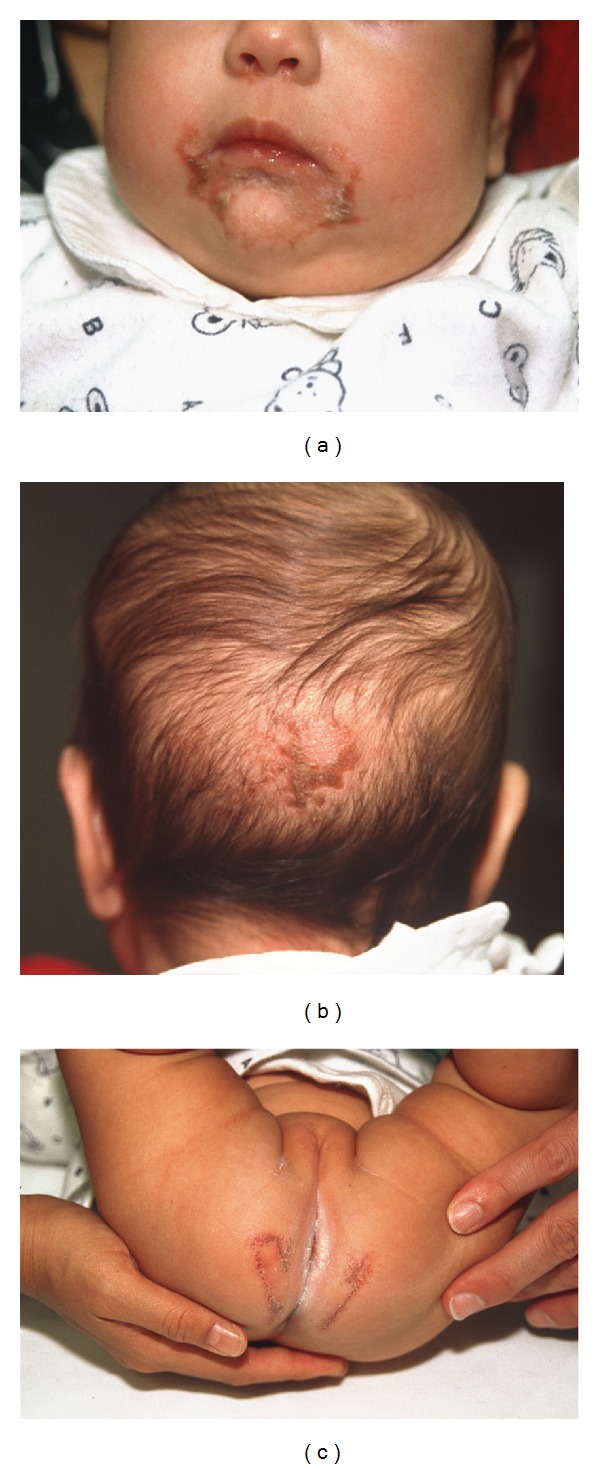
*Skin lesions*. Skin lesions in a perioral region (a), on the head (b), and in the anal region (c).

**Figure 3:**
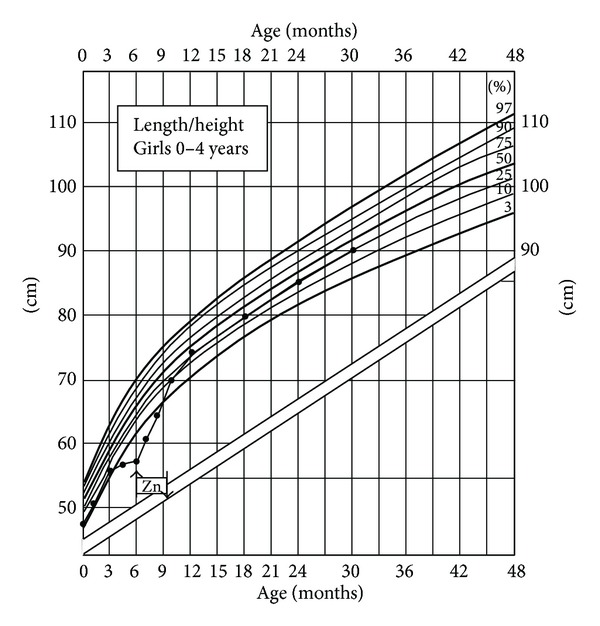
*Growth chart of the patient*. The solid circles indicate the length measurements. Percentiles are shown on extreme right. The arrows pointing up and down indicate the beginning and the end of the Zn^2+^ supplementation therapy.

Following an uneventful pregnancy the baby girl (II.3) was born at term (birth weight 3210 g, 50th centile; birth length 49 cm, 50th centile) [15] (Figure 1). The mother is of Philippine origin and the father of Swiss origin. The family history is uneventful and no genetic diseases are reported. The postnatal adaptation was normal. However, at the age of 6 months the infant was presented to the outpatient clinic of dermatology in the University of Bern, Switzerland. The clinical examination revealed an otherwise healthy, exclusively breast-fed 6 months old infant presented with a 3 weeks history of increasing skin problems, abdominal cramps, and diarrhoea with no obvious malnutrition. The skin lesions involving the face in a perioral distribution (Figure 2(a)), head (Figure 2(b)), and in the anal area (Figure 2(c)) appeared like an acrodermatitis enteropathica while the analysis of serum revealed a significantly low Zn^2+^ level (Table 1) and decreased level of alkaline phosphatase. Serum Zn^2+^ level in the mother was measured normal, while Zn^2+^ concentration in mother's breast milk was 0.12 mg/kg, which was significantly lower than the normative values (Table 1). Hence, all the clinical parameters were in line with the diagnosis of TNZD. In addition, while analysing length, weight, and length velocity of the infant, a stunted growth (IGF-I and IGF-BP3 levels at −2.3 SDS, −2.9 SDS, resp.) and a failure to thrive became obvious starting at the age of 3 months and progressed further until the age of 6 months, at the time when Zn^2+^ supplementation was started (Figure 3). At that point the infant also presented with a length that significantly dropped below the 3rd centile on the growth curve (Figure 3). An oral Zn^2+^ supplementation therapy (administered p.o, 4 mg/kg/day, as zinc sulphate heptahydrate) initiated and thereafter led to disappearance of all the clinical symptoms within the next four weeks.

### 2.2. DNA Isolation

For the genetic studies, written informed consent was obtained from both parents. Genomic DNA was isolated from peripheral leukocytes of the affected subjects using the QIAamp blood extraction kit (Qiagen AG, Basel, Switzerland) and used as a template for analysis of the *ZnT2* gene. The concentration of each sample was determined by measuring the optical density of the purified DNA at 260 and 280 nm.

### 2.3. Amplification and Sequencing of Genomic DNA

The genomic organization of human *ZnT2 *was determined from published internet data (http://www.ncbi.nlm.nih.gov/), and each one of the 8 coding exons was amplified by PCR amplification using established primers [10]. For PCR, approximately 100 ng of genomic DNA was used as template in a PCR SuperMix High Fidelity (Invitrogen, UK) in a total reaction volume of 50 *μ*L. PCR was carried out in TGradient Thermoblock (Biometra, Germany) under the following conditions: 33 cycles with an initial denaturation step at 94°C for 2 min, thereafter 94°C for 45 s, annealing at 60°C (for exons 1), 55°C (for exon 2 and 4), 61°C (for exon 3), 59°C (for exon 5, 6, 7), 57°C (for exons 8) for 45 s, and with an extension of 72°C for 1 min. Amplification was completed with an additional extension step at 72°C for 5 min. Negative controls included no template control. PCR products were separated on 1.5% agarose gel and stained with ethidium bromide. The bands corresponding to each specific PCR product were purified with QIAquick PCR Purification Kit (Qiagen AG, Basel, Switzerland) and sequenced on an ABI 373 automated DNA sequencing system (Applied Biosystems). The sequences were confirmed by re-PCR and resequencing from the genomic DNA of both strands.

## 3. Results

### 3.1. Identification of a Heterozygous G87R *ZnT-2* Mutation

**Figure 4:**
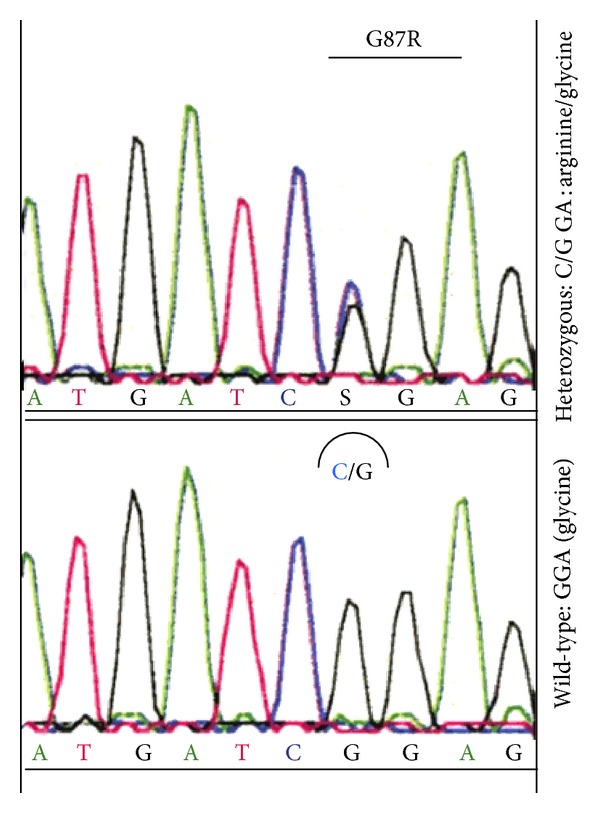
*Heterozygous G87R mutation in the ZnT2 (SLC30A2) gene*. At position 87 of the *ZnT2 (SLC30A2)* gene, a heterozygous mutation G > C (Gly > Arg) is depicted.

We identified a heterozygous G87R* ZnT2 *mutation in the mother (I.1) as well as in the baby/girl (II.3) (Figures 1–4). To know that II.3 does carry the mutation is very much of importance for her possible life as a breastfeeding mother. This mutation has been previously reported and studied in most detail at the functional level [10]. In this study as well in a previous report the authors showed that a functional inactivation of the *ZnT2* is the underlying cause of TNZD [10, 11]. 

### 3.2. Impairment of Growth and Development

The aim of our study, however, was to focus on the impact of length and length velocity in a Zn^2+^-deficient environment. Although the mechanisms are not well studied, it is well known and accepted that prolonged Zn^2+^ deficiency during infancy as well as childhood is not a negligible cause for stunting growth and failure to thrive [1–5]. 

### 3.3. Effect of Zn^2+^ Supplementation on Growth

As demonstrated in Figure 3, the oral Zn^2+^ supplementation therapy (administered p.o, 4 mg/kg/day, as zinc sulphate heptahydrate) resulted after 4 months in a complete catch-up growth of the child as well as normalized the IGF-I and IGF-BP3 levels (Table 1). Therefore, a sufficient Zn^2+^ serum concentration in this infant seems also to be of crucial importance for GH secretion and, thus, normal growth and development. 

### 3.4. Zn^2+^ Transporters and GH Secretion

By Inoue et al., *ZnT5*-null mice, as the result of crossing between heterozygous mice according to Mendelian expectations, were cloned and provided some *in vivo* data about the phenotype caused by the complete deletion of only one zinc transporter, namely, ZnT5 [16]. *ZnT5*-null mice displayed abnormal bone development, loss of weight, and lethal, male-specific, cardiac arrhythmia. Interestingly, these mice presented with significantly impaired growth when compared to the wild-type animals and with a high degree of osteopenia due to systemic decrease in bone density as the results of the reduced activity of osteoblasts [16]. Although the authors did not focus on growth, it has been nicely described and depicted in this study [16]. Further, Robinson et al. used the advantage of enhanced green fluorescent protein (eGFP), which when expressed from cell-specific promoters in transgenic animals, allows identification of specific cell types *in situ* and provides a fluorescent tag for their isolation and analysis, using fluorescence-activated cell sorting (FACS) technique [17, 18]. Therefore, eGFP was targeted to the secretory granules of pituitary GH-producing cells in transgenic mice (GH-eGFP transgenic mouse) [18, 19], followed by the FACS sorting of somatotrope cells (eGFP^+^ cells). Analysis of specific gene expression patterns using microarray technique was performed, and relative expression data of all zinc transporters assessed (Table 2) revealed the expression of ZnT5 to be the strongest in somatotropes. Hence, these data suggest high involvement of ZnT5 in the processes of GH storage and secretion.

## 4. Discussion

### 4.1. Zn^2+^ Deficiency

The initial main symptoms of mild Zn^2+^ deficiency are growth faltering as well as anorexia. Further prolonged and/or severe zinc deficiency presents with dermatitis and alopecia and is often expressed in growth impairment as well as neuropsychological alterations [20]. Infants are particularly vulnerable to Zn^2+^ deficiency, as they require large amounts of Zn^2+^ for their normal growth and development. Although term infants are born with an important hepatic zinc storage, adequate zinc nutrition of infants mostly depends on breast milk (especially in the first 3 months of lactation) or milk formula feeding, which contains an adequate amount of Zn^2+^ to meet the infants' requirements [6]. However, there are several causes for Zn^2+^ deficiency during infancy. First, it may be a result of deficient nutrition due to low level of Zn^2+^ in the breast milk or to consumption of food that is poor in Zn^2+^ bioavailability, or second, it is associated with distinct genetic disorders in Zn^2+^ metabolism [10, 11, 13, 21]. One of those genetic defects is associated with mutations of the intestinal Zn^2+^-specific transporter *Slc39a4/Zip4* [12, 13], which is responsible for Zn^2+^ absorption in the small intestine and when mutated leads to a rare, autosomal recessive disease called acrodermatitis enteropathica (AEZ) (OMIM number 201100). AEZ manifests in impaired intestinal Zn^2+^ absorption; hence, patients harbouring AEZ require lifelong zinc supplementation [14, 22]. Without therapy, plasma Zn^2+^ concentration and serum alkaline phosphatase, as well as urinary excretion of Zn^2+^, are very low [23]. Another genetic defect is associated with a mutation within *Slc30a4/ZnT4* and causes a reduced Zn^2+^ incorporation into mother's milk [11, 21]. Mice homozygous for a *ZnT4* mutation are known as lethal milk mice (lm^−^/lm^−^ mouse) producing milk, which is Zn^2+^ deficient (OMIM number 602095) [24]. As this phenotype in mice mirrors TNZD (OMIM number 608118) in breast-fed infants [7–9] Michalczyk et al. investigated whether changes in the *ZnT4* gene are responsible for reduced Zn^2+^ in breast milk in human in two unrelated mothers with low Zn^2+^ milk levels whose babies had developed Zn^2+^ deficiency. Their findings suggested that the lm^−^/lm^−^ mouse is not the corresponding model for the human Zn^2+^ deficiency condition [25]. Finally, TNZD in humans was found to be associated with mutations in *SLC30A2/ZnT2. *Gene knockdown of *ZnT2* in mammary epithelial cells reduced Zn^2+^ secretion, suggesting a role for this transporter in Zn^2+^ secretion from this cell type [11]. *ZnT2* was also found to be upregulated and relocalised to vesicles after exposure of mammary epithelial cells to prolactin. Similarly, mammary gland *ZnT2* was upregulated and relocalised to the luminal membrane in lactating rats when plasma zinc increased during lactation [26, 27]. Heterozygous H54R and G87R mutations in *ZnT2* were recently identified in women presenting with a Zn^2+^-deficient milk [10, 11]. As there is no impairment in Zn^2+^ uptake in the gut in affected babies, their infants, consequently, developed TNZD that was resolved after oral Zn^2+^ supplementation [10, 11]. As previously reported by Lasry et al. [10] we identified and characterized a heterozygous G87R mutation in *ZnT2*  leading to production of Zn^2+^-deficient milk in a mother originated from the Philippines; as a result, their exclusively breast-fed infants developed TNZD with low Zn^2+^ blood levels that resolved upon Zn^2+^ supplementation. As far as the function is concerned, Lasry et al. showed that the G87R mutation is a loss of function mutation and they provided, therefore, the first evidence for the dominant inheritance of heterozygous *ZnT2* mutations via negative dominance due to homodimer formation [10].

### 4.2. Impact of Zn^2+^ on Growth Hormone Secretion [28]

The *growth hormone-1* (*GH-1*) gene is mainly expressed as a major 22 kDa isoform in somatotrope cells of the anterior pituitary gland. After being translated, GH protein passes throughout the regulated secretory pathway where it gets packed and stored in concentrated forms in secretory granules enabling a regulated release in circulation upon GHRH stimulation [29, 30]. 

Zn^2+^ is considered as the second most abundant “trace” metal in the human body which is required for numerous cellular mechanisms like DNA synthesis, protein synthesis, cell growth, and division [31] as well as for many physiological processes like immune function [32] and reproduction [33, 34]. Hence, cellular Zn^2+^ homeostasis and dynamics are tightly regulated and maintained by various Zn^2+^ transporters responsible for transporting these high charge density ions across cellular membranes and various intracellular organelles [35, 36]. Over two decades ago, a high concentration of Zn^2+^ was reported to be localized mostly in the Golgi complex and GH-containing secretory granules of rat anterior pituitary cells [37], suggesting in that way, an important role of Zn^2+^ in the regulated secretory pathway of GH. During the process of secretory granule biogenesis, self-association (aggregation) of a hormone destined for secretion facilitates its storage in granules in fairly high amounts, and in the case of GH, it occurs in the presence of Zn^2+^ [38]. 

### 4.3. The Biogenesis of GH Secretory Granules Begins with Zn^2+^-Mediated GH Aggregation at Acidic pH in the *trans*-Golgi Lumen

The complex process of secretory granule biogenesis begins with aggregation of proteins (hormones) destined for secretion to form dense cores of granules composed of large insoluble aggregates. Upon appropriate stimulation, aggregates are released into the bloodstream leading to a burst of hormone on a time scale much faster than it could be achieved from increased synthesis. 

Protein aggregation takes place in the lumen of *trans*-Golgi layer where specific environmental factors seem to play an important role in inducing this process. In fact, apart from specific pH requirements [39, 40], aggregation of GH apparently requires high amounts of divalent cations like Zn^2+^ [37]. 

A step further towards unravelling the role of Zn^2+^ in storage of GH in secretory granules came with the study reporting that two Zn^2+^ associates per GH dimer in a cooperative fashion through binding at high-affinity residues in GH (His18, His21, and Glu174) [41]. Replacement of these residues with alanine caused reductions of dimeric GH formation as demonstrated by size-exclusion chromatography and sedimentation equilibrium analysis. In addition, the data presented also demonstrate that Zn^2+^ binding to GH would enhance stability of the stored form and that Zn^2+^-GH complex was more stable to denaturation when compared to monomeric GH ultimately proposing that Zn^2+^-GH dimer may be the main storage form in the secretory granules [41].

The potential contribution of high-affinity Zn^2+^-binding residues in GH to the pathogenic mechanisms involved in dominantly transmitted isolated GH deficiency type II (IGHD II) was further studied by Iliev et al. [42]. The production and extracellular secretion of *wt*-hGH transiently transfected in GH_4_C_1_ cells (rat pituitary tumour cells) were compared to that of GH mutants in which the amino acids that bind Zn^2+^ with high affinity were mutated to alanine in various combinations. When *wt*-hGH was coexpressed with any of the Zn^2+^-binding GH mutants, constitutive GH secretion (i.e., without stimulation) and intracellular production remained unaffected. Interestingly, each of the Zn^2+^-binding GH mutants (single, double, or triple mutants) singly expressed displayed about 50% lower extracellular secretion and intracellular production when compared to the *wt*-hGH suggesting possible role of these residues in GH stability. 

GH and PRL are two hormones that are structurally related, and therefore it is of no surprise that they display many similarities in the process of aggregation as reported in the study mentioned above. However, alanine mutation introduced at His27 in hPRL (topologically corresponding to His18 in hGH) resulted in H27A-PRL mutant reported not to bind Zn^2+^ [43]. Interestingly, even without the high-affinity Zn^2+^ binding site, H27A-PRL is still able to aggregate in the presence of Zn^2+^ with parameters similar to aggregation of *wt*-PRL [40]. Hence, these data suggest that PRL and GH do not behave similarly in the presence of Zn^2+^ and that PRL does not form dimer under the conditions that GH does, indicating that the dimer is unlikely to be the storage form of PRL in secretory granules. Zn^2+^ binding to human PRL and GH can occur through histidine residues (the high-affinity binding sites) [41, 43] or through glutamate, aspartate, and glutamine residues (the low-affinity binding sites) [44]. Acidic pH in the *trans*-Golgi lumen where the process of aggregation occurs leads to protonation of His residues preventing their binding to Zn^2+^. Therefore, it is more likely that Zn^2+^ binding to glutamate and aspartate residues (low-affinity binding) of PRL facilitates the formation of PRL oligomers as the storage form in dense cores of secretory granules.

Finally, as mentioned earlier Zn^2+^ binding to GH through high-affinity binding sites is proven to be necessary for the formation of GH dimers, but whether this is the final storage form of GH in secretory granules still remains to be elucidated. Alternatively, additional intramolecular cross-linking might occur through low-affinity Zn^2+^-binding with amino acids other than histidine (as described above for PRL) enhancing in that way GH aggregation and storage in secretory granules.

### 4.4. Zinc Transporters Mediate Zn^2+^ Dynamics in the Early Secretory Pathway and Might Play an Important Role in the Formation of GH-Containing Secretory Granules

Out of all proteins synthesized in eukaryotic cells approximately one-third is targeted to the secretory pathway [45] and the first compartment encountered along their road towards secretion is the ER. Together with Golgi complex, ER comprises the early secretory pathway, which plays the key role in regulating the folding, assembly, and transport of newly synthesized proteins and modification and trafficking during the secretory process. There are estimates that between three and ten percent of all proteins in mammalian genomes bind Zn^2+^ [46], and many zinc-dependent proteins pass through the secretory pathway on their way to other compartments within the cell (e.g., vacuole, lysosomes) or prior to their secretion. Due to its high charge density, Zn^2+^ requires transporters to move it across the cellular membranes and in and out of each of the organelles participating in the regulated secretory pathway (ER, Golgi complex, and secretory granules). More than 20 zinc transporters identified and characterized up to date, classified into two families (Slc30a/ZnT and Slc39a/Zip), reflect the complexity and importance of maintaining cellular Zn^2+^ homeostasis and dynamics. The role of ZnTs is to reduce intracellular Zn^2+^ by transporting it from the cytoplasm into various intracellular organelles and by moving Zn^2+^ into extracellular space. Zips increase intracellular Zn^2+^ by transporting it in the opposite direction. Thus the coordinated action of both is essential for the maintenance of Zn^2+^ homeostasis in the cytoplasm, and accumulating evidence suggests that this is also true for the secretory pathway.

### 4.5. Conclusion: Where the Mother's Milk Meets the Baby's Growth

Having discussed the importance of Zn^2+^ as well as their individual intracellular transporters it comes without any surprise that an adequate Zn^2+^ concentration in the child's plasma is of high importance for normal growth and development [1, 3, 28, 47–49]. 

## Figures and Tables

**Table 1:** Laboratory data of the child (II.3) and mother.

	Child			Mother
	Before Zn^2+^ supplementation	After 1 month on Zn^2+^ supplementation	Normative values	
Zn^2+^ in serum (*µ*mol/L)	2.3	14.6	9–21	12.2 (11–18)
Alkaline phosphatase (IU/L)	73	465	96–336	88 (36–108)
IGF-I (ng/mL)	10 (−2.3 SDS)	62 (0 SDS)	18–146	
IGF-BP3 (mg/L)	0.89 (−2.9 SDS)	2.95 (+0.5 SDS)	1.19–3.81	
Zn^2+^ in breast milk (mg/kg)				0.12 mg/kg (0.2–0.76^(6)^)

**Table 2:** Relative expression data of zinc transporters in GFP-sorted somatotrope cells from GH-eGFP transgenic mouse.

Zn transporter	Relative expression	Gene bank	Significance
Slc30a1	1.7235773	NM_009579	0.76
Slc30a3	2.1382113	U76007	0.66
Slc30a4	7.08943	NM_011774	0.18
**Slc30a5**	**28.886179**	**NM_022885**	**0.028**
Slc30a6	4.1382113	AF233346	0.34
Slc30a7	9.601625	AF233322	0.12
Slc30a9	9.593495	BB117951	0.12

*P* < 0.05 is significant.
